# Morphological changes, nitric oxide production, and phagocytosis are triggered *in vitro* in microglia by bloodstream forms of *Trypanosoma brucei*

**DOI:** 10.1038/s41598-018-33395-x

**Published:** 2018-10-09

**Authors:** Katherine Figarella, Nestor L. Uzcategui, Stefan Mogk, Katleen Wild, Petra Fallier-Becker, Jonas J. Neher, Michael Duszenko

**Affiliations:** 1 Interfaculty Institute for Biochemistry, University of Tübingen, Tübingen, Germany; 2 Institute for Neurophysiology, University of Tübingen, Tübingen, Germany; 3 Institute for Anatomy, Central University of Venezuela, Caracas, Venezuela; 4 German Center for Neurodegenerative Diseases (DZNE), Tübingen, Germany and Department of Cellular Neurology, Hertie Institute for Clinical Brain Research, University of Tübingen, Tübingen, Germany; 5 Institute of Pathology and Neuropathology, University Hospital of Tübingen, Tübingen, Germany; 6 Faculty of Medicine, Tongji University, Shanghai, China

## Abstract

The flagellated parasite *Trypanosoma brucei* is the causative agent of Human African Trypanosomiasis (HAT). By a mechanism not well understood yet, trypanosomes enter the central nervous system (CNS), invade the brain parenchyma, and cause a fatal encephalopathy if is not treated. Trypanosomes are fast dividing organisms that, without any immune response, would kill the host in a short time. However, infected individuals survive either 6–12 months or more than 3 years for the acute and chronic forms, respectively. Thus, only when the brain defense collapses a lethal encephalopathy will occur. Here, we evaluated interactions between trypanosomes and microglial cells, which are the primary immune effector cells within the CNS. Using co-cultures of primary microglia and parasites, we found clear evidences of trypanosome phagocytosis by microglial cells. Microglia activation was also evident; analysis of its ultrastructure showed changes that have been reported in activated microglia undergoing oxidative stress caused by infections or degenerative diseases. Accordingly, an increase of the nitric oxide production was detected in supernatants of microglia/parasite co-cultures. Altogether, our results demonstrate that microglial cells respond to the presence of the parasite, leading to parasite’s engulfment and elimination.

## Introduction

Human African Trypanosomiasis (HAT), caused by the protozoan parasite *Trypanosoma brucei* (*T. brucei*), represents a relevant threat to people living in the sub-Saharan region in Africa. It is estimated that up to 70 million people are at risk of contracting HAT^1^. Depending on the *T. brucei* subspecies involved, HAT manifests itself in two different forms; an acute form, in the case of *T. brucei rhodesiense* or a chronic form, in the case of *T. brucei gambiense*. The survival time of an infected individual is estimated to be either 6–12 months or around 3 years for the acute and chronic forms, respectively^2,3^. After a blood meal of an infected tsetse fly, the parasite remains for some time in the skin before it reaches lymph and blood vessels to initiate infection^4^, which evolves in two stages. The first (haemolymphatic) stage is characterized by the presence of parasites in blood and lymph. During this period patients typically show unspecific symptoms that complicate an early diagnosis and appropriate treatment. A very unfortunate situation, as medicals unable to penetrate the blood-brain barrier (suramin, pentamidine) are far less toxic than those entering the brain (melarsoprol B, eflornithine). To initiate the second, so-called meningoencephalitic stage, parasites cross physical and immunological barriers to invade the brain. This is the most devastating phase of the infection. The clinical manifestations include severe sleep/wake disturbances, the reason why the disease is alternatively called sleeping sickness, neurological and psychiatric disorders, coma, and death, if left untreated^5–8^. Mechanisms underlying invasion of the brain by *T. brucei* are not totally understood. Some authors found experimental evidences for penetration of the blood brain barrier^9–12^, while others showed that trypanosomes penetrate the blood-CSF barrier and settle within the meninges^13–15^. In the latter case, translocation across the pia mater and the Virchow-Robin space would be the likely route to enter the neuropil. Interestingly, however, trypanosomes injected directly into the neuropil did not induce infection^13^ and disappeared from the site of injection within 24 h^14^.

Since immunosuppression favours *T. brucei’s* invasion into the CNS, the host immune response plays a pivotal role in the development of the observed neuropathology^12^. Within the brain parenchyma, microglia are the immune cells accountable for the defense against pathogens that target the CNS. Under physiological conditions microglia show a “resting or surveilling” phenotype. They have a small soma with fine, motile, and complex ramified cellular processes, which constantly monitor their microenvironment^16^. Any perception of brain homeostasis disturbance indicating possible danger, as infection, trauma, etc., generates a rapid morphological change, the so-called “activation state”, which is characterized for profound transformations in the microglial metabolism and cell shape^17^. Phenotypically, they show a larger cell body and reduction of cellular processes, characteristically microglia become spherical or amoeboid cell type, which allows them to move to the lesion and to phagocyte infectious agents or repair the damage by engulfing neuronal debris^17,18^. Beside their direct role in the innate immunity to foreign invaders, they also participate in coordinating the trafficking and recruitment of other immune cells from the periphery to the CNS. However, the role of microglial cells in HAT has so far not been investigated, although microglial nodules have been clearly documented in human pathological studies^19^. In this work, we evaluated the interactions between microglia and trypanosomes *in vitro* and discuss the possible implications of our findings for the disease control during the meningoencephalitic stage.

## Results

### Creation of mutant parasites with defective propulsive motility

**Figure 1:**
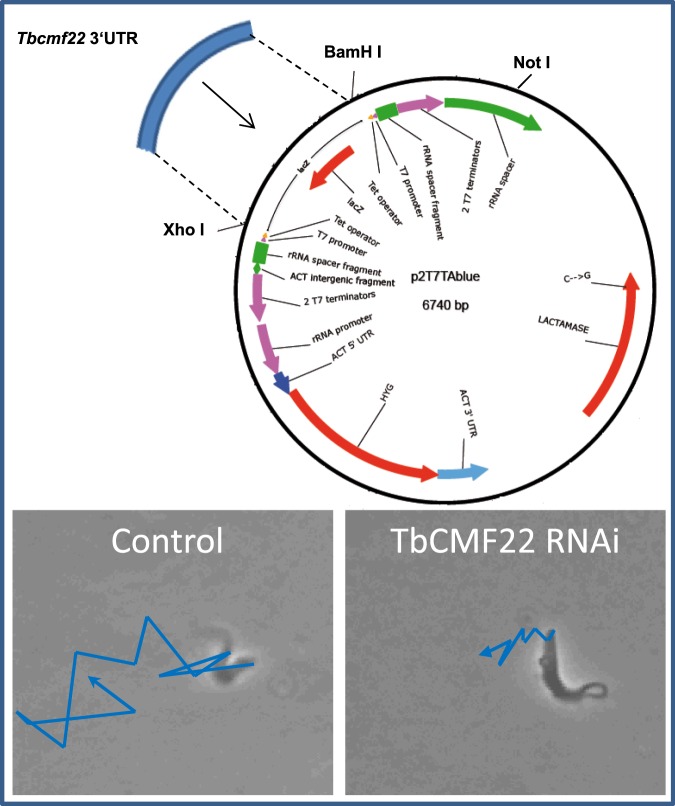
Translocation capability of TbCMF22 knockdown trypanosomes. Translocation was video-monitored using a Zeiss Z1 AxioObserver inverted microscope attached to a video camera. Upper: plasmid map indicating restriction sites used for amplification (BamH I and Xho I), and the restriction site used for linearization prior to transfection (Not I); the LacZ gene was removed during amplification. Lower: translocation of control and TbCMF22 RNAi cells after 72 hours RNAi induction. Blue lines represent translocation, as measured by position changes of the flagellar pocket from the parasite, within two seconds.

Bloodstream trypanosomes are very motile organisms that physiologically swim freely in blood or under *in vitro* conditions in culture medium. Within tissue, however, parasites experience translocation resistance due to the surrounding environment. It has been proposed that swimming velocity in blood contributes to the evasion of the immune system including clearance by macrophages^20^. Interestingly, during experimental infection, trypanosomes have been found in several tissues, where morphological evidences for parasite phagocytosis by macrophages were observed^14^. In addition, *in vitro* experiments using wildtype and motility-defective trypanosomes showed that the latter is significantly better phagocytosed by macrophages than the former (data not shown). Therefore, to analyse interactions between microglial cells and trypanosomes, a mutant *T. brucei* strain with a defective propulsive motility, was used for co-incubation with acutely isolated adult microglia from mouse brain. The mutant *T. brucei* strain showing a highly reduced translocation capacity, without losing the flagellum motility, was created according to Nguyen *et al*.^21^ by knocking down TbCMF22 transcripts, targeting the 3′UTR region of the Tbcmf22 gene. TbCMF22 is a protein member of the components of motile flagella (CMF), which is responsible for propulsive cell motility and appears to function in flagellar beat regulation^21^. CMF22 deficient trypanosomes would mimic the limited displacement capacity behaviour within the brain tissue. A specific p2T7Ablue-TbCMF22RNAi construct (Fig. 1, upper panel) was electroporated into SMB bloodstream forms and transfected parasites were selected with hygromycin. RNAi induction was performed by addition of tetracycline to the culture medium and interference was confirmed by the diminished motility of mutant parasites (Fig. 1, lower panel), as previously reported^21^. CMF22 deficient parasites grew normally and their proliferation rate under *in vitro* culture conditions resembled that of control parasites showing generation doubling times of 10.7 h instead of 8.3 h, respectively. Moreover, mutant parasites retained their infectivity, as an intraperitoneal inoculum of 1 × 10^7^ CMF22 deficient parasites produced infection in C57BL/6 mice (results not shown).

### Microglia phagocytose trypanosomes *in vitro*

**Figure 2:**
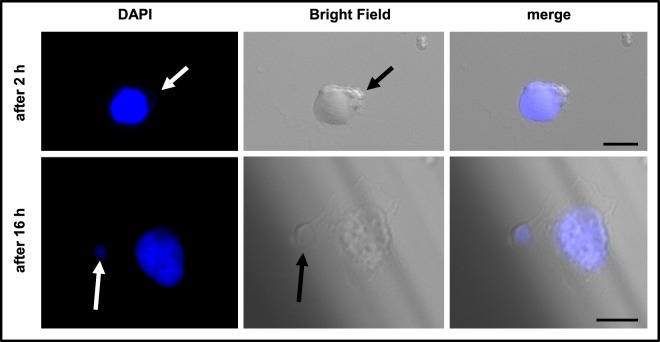
Interaction between microglia and trypanosomes after different incubation times. Pictures show parasites (indicated by arrows and recognized by nucleus and kinetoplast staining) in direct contact with microglia. Note that parasites are firmly attached to microglia because they remain bound after removing of supernatants and washing wells with fresh buffer. In the panel below (16 h) fluorescence signals are seen in the cytoplasm of microglia. Images were taken with the Zeiss Z1 AxioObserver inverted fluorescence microscope. The microglia:parasite ratio was 1:30. Analysis was performed with the AxioVision System Software. Bars represent 10 μm.

Although microglia isolation from adult brain is a challenging and laborious time-consuming task with low yields, we decided to work with these primary cell cultures since this is the most reliable approach to evaluate functions of these cells *in vitro*. Immortalized microglia-derived cell lines, represent an unlimited source of cells, however, they are produced by the expansion of one clone, losing important characteristics and markers from the original population. Due to their rapidly dividing capacity immortalized microglia change significantly their characteristics over time^22^. In our approach, microglia were isolated from adult C57BL/6 mice by fluorescence activated cell sorting (FACS; see materials and methods). Accurately isolated microglia were challenged with the TbCMF22-RNAi mutant strain for different incubation times, then fixed with paraformaldehyde and stained with DAPI. Using phase contrast and fluorescence microscopy, we could observe trypanosomes in direct contact with microglial cells already after 2 hours of co-incubation. Interestingly, parasites were firmly attached to microglial cells, as they remained bound after aspiration of supernatants and washing adherent microglia twice with fresh buffer (Fig. 2, upper panel). After longer incubation times (16 h) fluorescence signals were also detected within the cytoplasm of some microglia, suggesting parasite engulfment or uptake (Fig. 2, lower panel).

**Figure 3:**
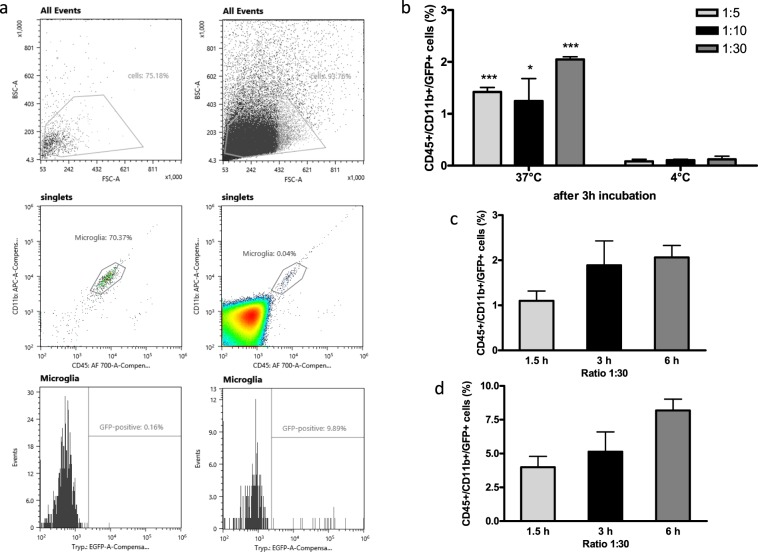
Phagocytosis analysis by flow cytometry. (**a**) Dot plots and histograms showing the CD45^int^/CD11b^high^ double-labelled population corresponding to microglia and the CD45^int^/CD11b^high^/eGFP^+^ triple-labelled population representing microglia that have phagocytosed parasites. Left and right panels show microglia alone and microglia co-incubated with parasites for 6 h, respectively. (**b**) Percentage of triple-positive microglia after 3 h of co-incubation at 37 °C and 4 °C, respectively. The microglia to parasites ratios were 1:5, 1:10, and 1:30, respectively. (**c**) Time-dependence of phagocytosis evaluated at a 1:30 ratio and 37 °C in co-cultures, in which unbound parasites or detached microglia were washed out. (**d**) Bars illustrate the percentage of triple labelled cells in supernatants from cultures in C (mean ± S.D. of three individual cultures measured at each condition). The data were processed using the FlowJo software (FlowJo, LLC, Ashland, Oregon, USA). Asterisks represent significant differences, p < 0.001 (***) and p < 0.05 (*).

For confirmation and further analysis, phagocytosis was also evaluated applying an established protocol, a flow cytometry-based microglial phagocytosis assay, with which quantitative, reliable, and objective measurements can be performed^23^. For this purpose, the TbCMF22-RNAi mutant strain was again transfected to express the eGFP gene. Sorted microglia were incubated with TbCMF22-RNAi-GFP parasites under different conditions (see material and methods) and microglia containing phagocytosed parasites were quantified by flow cytometry. The existence of a triple-labelled population (CD45^int^/CD11b^high^/eGFP^+^) in experiments at 37 °C and its almost complete absence at 4 °C indicate that microglia ingest trypanosomes by phagocytosis (Fig. 3). Co-incubations at 4 °C were performed to differentiate between phagocytosis and an unspecific cell adhesion, since the former does not occur at low temperatures^24^. Data acquired from experiments performed at 4 °C, in all conditions evaluated, showed a negligible triple labelling of below 0.2% (Fig. 3b), assuring that the experimental observation described above corresponds indeed to phagocytosis. At 37 °C, the highest percentage of phagocytosis was obtained at a 1:30 microglia:parasite ratio, although phagocytosis was also detected at a 1:5 ratio (Fig. 3b). Phagocytosis occurs rapidly, as triple-labelled microglial cells were detected as early as 1.5 hours after addition of trypanosomes (Fig. 3c,d). Noteworthy, most of the triple-labelled microglia appeared in the culture supernatant, i.e., 3.99 ± 1.61% and 8.19 ± 1.67% in the supernatant vs. 1.02 ± 0.42% and 2.06 ± 0.46% in adherent cells after 1.5 h and 6 h, respectively, indicating that during or after phagocytosis microglia undergo molecular changes regarding their adherent properties.

### Parasite-challenged microglia show ultrastructural alterations compatible with activation and phagocytosis

**Figure 4:**
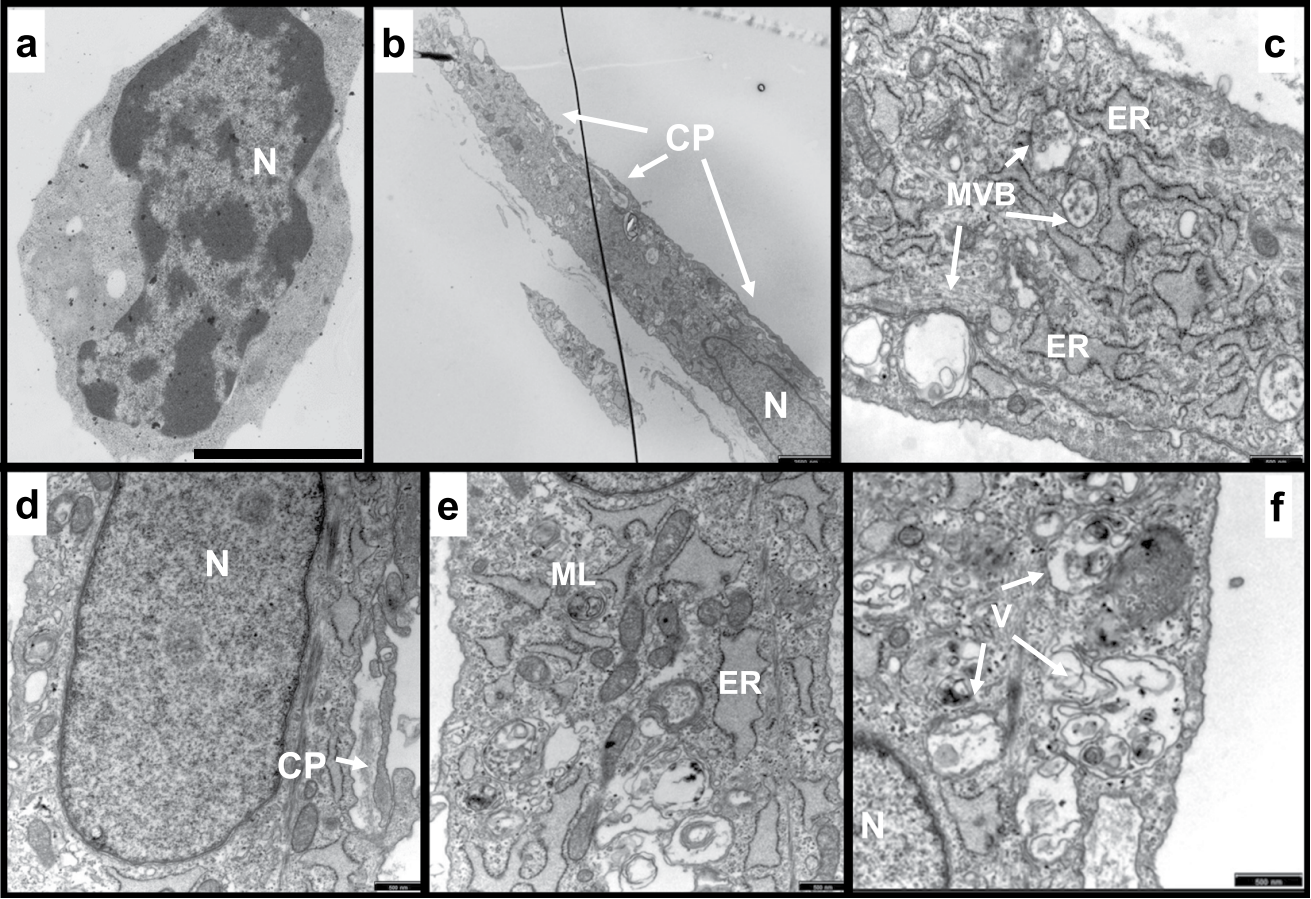
Ultrastructural analysis of CD45^int^/CD11b^high^/eGFP^+^ microglia. (**a**) Typical morphology of control microglia. (**b**–**f**) Main ultrastructural changes observed in CD45^int^/CD11b^high^/eGFP^+^ cells after 6 h of co-incubation with trypanosomes at 37 °C. Note alterations in the nucleus/cytoplasm ratio in B with respect to A. Abbreviations: Nucleus (N), cytoplasmic protrusions (CP), multivesicular bodies (MVB), endoplasmic reticulum (ER), myelin-like structures (ML), vacuoles (V). Bars in A and B represent 2.5 µm; bars in C-F denote 0.5 µm.

Triple-labelled as well as control microglial cells were sorted and prepared for transmission electron microscopy. Here, microglia not challenged with parasites (control condition) displayed prominent nuclei, scarce cytoplasm and characteristic organelles, i.e. typical characteristics of acutely isolated cells in culture (Fig. 4a). In contrast, micrographs of CD45^int^/CD11b^high^/eGFP^+^ microglia showed evidence of activation as well as typical structures suggestive of phagocytic activity (Fig. 4b–f). In fact, most of the triple-labelled cells displayed an enlarged cytoplasm, as judged by the nucleus/cytoplasm ratio (Fig. 4b), endoplasmic reticulum expansion (Fig. 4c,e), and the presence of multivesicular bodies (Fig. 4c). Another frequent observation was the occurrence of cytoplasmic protrusions (Fig. 4b,d) and vacuoles containing degraded cellular material (Fig. 4e,f), which are characteristic for phagocytic processes. Moreover, myelin-like structures (i.e., concentric membranes), an indicator of increased organelle recycling and degradation of membranes by autophagy, were also observed (Fig. 4e).

### Induction of nitric oxide production in parasite-challenged microglia

**Figure 5:**
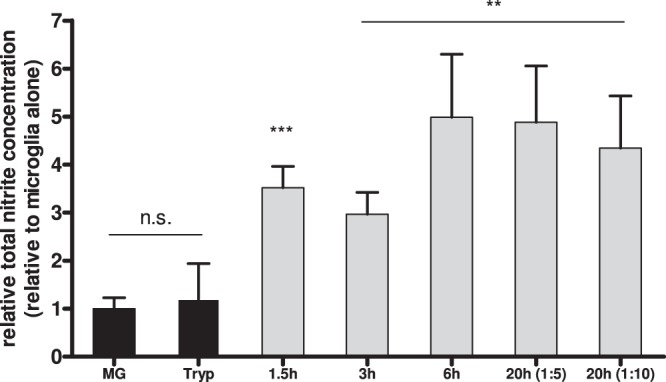
Nitric oxide levels in supernatants of microglia-parasite co-cultures. Microglia were maintained in culture alone or together with trypanosomes at a 1:30 ratio, unless otherwise specified. After indicated time points, supernatants were collected and prepared for nitric oxide determination using the Total Nitric Oxide Assay from R&D Systems and following the manufacture’s recommendations. Total nitrite concentration was normalized to that obtained for microglia alone to achieve fold increase in each case. ns: not statistically significant. MG: microglia. Tryp: Trypanosomes. Asterisks represent significant differences, p < 0.001 (***) and p < 0.01 (**).

All morphological, ultrastructural, and functional changes described above are indicative of cell activation. To evaluate this phenomenon further and to obtain evidence for an inflammatory response, the concentration of nitric oxide (NO) was determined in the supernatant of microglia cultures and trypanosome-challenged microglia. NO and reactive oxygen species are indicative of activated microglia^25^ and are key cytotoxic weapons used by the innate immune system to eliminate parasites such as trypanosomes^26^. It is not an easy task to measure NO directly in culture media, since this compound is a gaseous free radical with a half-life of just a few seconds. Therefore, we have used a method to determine more stable NO derivative compounds (nitrite and nitrate), which provides an indirect estimation of the NO concentration (see material and methods). Under our experimental conditions, increased NO production could be detected after 1.5 hours and throughout the experiments in supernatants of microglia/parasites co-cultures. In all cases where microglia were exposed to trypanosomes, a significant increase of the NO concentration (-measured as total nitrite-) was detected, which was up to 5 times higher (10.57 ± 2.53 µmol/L after 20 h incubation at a 1:5 ratio) than control values measured for microglia alone (1.45 ± 0.33 µmol/L). In contrast, in microglia or in parasite axenic cultures the NO concentration did not increase, but remained stable on a low level within the 20 hours evaluated (Fig. 5).

## Discussion

Research carried out over the last two decades has shown that microglia represent an important cellular component involved in the development of many brain pathologies including infectious diseases^16^. As resident immune cells of the CNS, microglia play a critical role in the crosstalk not only among cells within the brain but also with systemic immune cells that are responsible for combating infections outside the CNS^27^. In case of *Trypanosoma brucei* infections, the condition of the host’s immune system is decisive for the development of a cerebral pathology, which is the most dangerous complication of the disease. However, the immune response plays sometimes an ambiguous role as it can act not only against but also in favour of a *T. brucei* neuroinvasion. The routes and molecular mechanisms used by the parasite to enter the meninges and the brain parenchyma remain to be fully clarified^28,29^, but it has been described that some immunological factors promote neuropil invasion. Mice deficient of IFN-γ or its receptor have reduced parasite penetration of the neuropil albeit increased parasitemia^9^. Treatment with minocycline, an antibiotic with anti-inflammatory activity, impeded the passage of both T cells and trypanosomes into the brain^10^. Furthermore, administration of the counter-inflammatory cytokine IL-10 diminish the entrance of *T. brucei* into the brain parenchyma and the subsequent neuro-inflammatory pathology^28^. In general, it seems that a systemic inflammatory response favours neuroinvasion and therefore the development of CNS pathology. Hence, it is mandatory to analyse in detail the complex interplay of factors involved in the immune system during the course of a *T. brucei* infection, particularly the response of microglial cells. Once parasites have reached the brain parenchyma, they should be killed and removed most likely by microglia. Interestingly, in several experimental approaches injecting life trypanosomes directly into the neuropil it could be shown that parasites do not survive inside the brain parenchyma, but disappear within a couple of days^13,30^. However, phagocytosis of *T. brucei* by microglia has not been clearly shown until now.

In this study, we used primary adult microglial cells and *T. brucei* bloodstream forms with a diminished translocation capacity that were created to mimic the parasite’s limited mobility within brain tissue, to evaluate their interactions under *in vitro* conditions. Before phagocytosis begins, activation of the phagocytic cell must take place. As judged by our electron micrographic analyses, microglia activation after parasite challenge *in vitro* was clearly apparent. The analysis of morphological and ultrastructural features showed changes that have been reported in activated microglia undergoing oxidative stress caused by infections or degenerative diseases^31^. Similar changes in microglial morphology have also been observed by exposing them *in vitro* to different *Leishmania* species or Plasmodium-infected red blood cells^32,33^. Consistently, induction of NO production was detected in supernatants of microglia/*T. brucei* co-cultures, which may be originated by an increase of expression of inducible NO synthase (iNOS) from microglia. Accordingly, an increase of the NO concentration as a result of an enhanced activity of iNOS in neurons and glial cells (astrocytes and microglia), evidenced by immunohistochemical studies, was found in the brain of rats infected with *T. brucei* during the meningoencephalitic stage^34^. Moreover, it was demonstrated, at the transcriptional level, that astrocytes and microglia challenged with *T. brucei* increase the expression of iNOS^35^. Interestingly, the activation of microglia may have different pathological outcomes, since a correlation between microglia activation and the onset and progression of sleep anomalies in the brain of trypanosome-infected rats has been described^36^.

Recently, a rapid report showed an image of *T*. *brucei* inside a microglia from a particular cell line (CMH-5), without any further experiments, raised the question whether these parasites have the ability to invade and live within microglial cells^37^. *T. brucei* is considered an exclusively extracellular parasite; only few early studies have occasionally showed the presence of some African trypanosomes within mammalian cells, like choroid plexus epithelium and astrocyte-like cells^38–40^. In the light of the current knowledge, there are not enough evidences for establishing this new intracellular stage and, in accordance with our results, microglial cells seem not to be a possible host for the parasite.

Here, applying several approaches under different *in vitro* experimental conditions, we show clear evidence that microglial cells may play a key role in the clearance of the parasite from the neuropil as these cells were able to phagocytose *T. brucei* bloodstream forms efficiently. Our results showed that under all experimental conditions microglia were activated and able to interact with trypanosomes. Phagocytosis increased with both incubation time and parasite to microglia ratio, leading to the highest phagocytosis rate of 8.19 ± 1.67%, while unspecific binding was 0.12 ± 0.08%. Activation of microglial cells co-cultured with trypanosomes was manifested by profound morphological changes, a significant increase of NO release and the fact that most microglia involved in phagocytosis lost surface adherence and were thus detected in the supernatant, consistent with prior observations^41^.

Microglial phagocytosis of other protozoan parasites has been described previously. For example, it was demonstrated that *in vitro* microglia are able to ingest and destroy plasmodium-infected red blood cells^32^ and free-living *Acanthamoeba spp*.^42^, both causative agents of diseases affecting the CNS, inducing cerebral malaria and granulomatous amoebic encephalitis, respectively. In addition, microglial cells have a high phagocytic ability to eliminate several *Leishmania* species^43^. *Leishmania* are obligate intracellular parasites in vertebrate hosts and together with trypanosomes belong to the trypanosomatid family. Leishmaniasis only occasionally involves the CNS^33^ and, therefore, it has been suggested that microglia, due to their efficient surveillance and clearance of *Leishmania* parasites by phagocytosis, may play a key protective role against cerebral leishmaniasis^43^.

Although the importance of microglia in the defence of the CNS is well known and the devastating consequences of cerebral infection in HAT are obvious, until now there is scarce literature addressing this issue. At least, some evidence in line with the importance of *T. brucei* phagocytosis by microglia alluded in this study have been reported. In post-mortem samples of patients with African trypanosomiasis the presence of diffuse meningoencephalitis, diffuse microglial hyperplasia, proliferation of large astrocytes, and formation of microglial nodules was described by immunohistochemical observations^44,45^. Therefore, this study stresses the importance and opens up new avenues for understanding the cellular function of microglial cells in the meningoencephalitic stage of HAT. Taken together, our results demonstrate that *in vitro* microglia respond to the presence of the parasite by activation and phagocytosis. Microglial cells increase their NO production and change their morphology visibly by increasing cytosol extensions with irregular shapes and formation of pseudopodia. To what extend phagocytosis takes place under *in vivo* pathophysiologic conditions and how it can influence progression of the disease has to be evaluated in animal models. Elucidation of this fact may change our point of view with respect to microglia as a therapeutic target, modifying their activation pattern and favouring their protective action.

## Materials and Methods

### Parasites, media, and reagents

In this work the modified *Trypanosoma brucei brucei* strain SMB (single marker bloodstream) was used^46^. Parasites were cultivated in HMI-9 medium containing 10% inactivated foetal bovine serum (FBS) and penicillin-streptomycin under a 5% CO_2_ atmosphere at 37 °C. Freshly isolated microglia were maintained in DMEN medium without FBS in a CO_2_ incubator at 37 °C for at least 2 hours to allow adherence. Chemicals were purchased from Sigma unless otherwise stated. Antibodies were acquired from Biozol.

### Creation of mutant parasites with defective propulsive motility

SMB parasites were stable transfected by electroporation with the construct p2T7Ablue-TbCMF22RNAi, which contains a fragment from the 3′UTR region of the Tbcmf22 gene^21^ cloned in between the 2 T7 promotors using a homologous recombination approach according to the InFusion Cloning Kit® (ClonTech, Nederland) instructions. The plasmid was amplified using the forward primer 5′CGGATCCACTAGTTCTAGAGCGG′3 and the reverse primer 5′TTAACTCGAGGGGGGGCC′3 in order to remove the lacZ sequence. Amplification of the insert included, besides the insert specific sequence (underlined), a plasmid specific sequence important for homologous recombination. Positive clones were analysed by double digestion using XhoI and BamHI restriction enzymes and their identity was confirmed by sequencing. Parasites were then electroporated with 10 µg of the NotI linearized p2T7blue-CMF22RNAi construct and incubated for 5–8 days under hygromycin pressure (2.5 µg/ml) for selection. RNAi induction was performed by addition of 2 µg/ml tetracycline to the culture medium for 72 h. Mutant parasites were confirmed by their diminished translocation capacity as compared to control parasites bearing only the vector. To analyse the phenotype of induced parasites, the translocation capacity was video monitored using a Zeiss Z1 AxioObserver inverted fluorescence microscope.

### Primary microglia culture

Freshly isolated microglial cells were obtained from 4–6 months old adult C57BL/6 mice (Charles River) as previously described^47^. In brief, mice were anesthetized with Ketamine/Xylazine and perfused with PBS. Brains were dissected and cerebral hemispheres were dissociated in Hank’s balanced salt solution containing 50 mg/mL glucose and 0.02 mg/mL Dnase I. Cells were separated on a Percoll gradient and double stained using Alexa Fluor® 700 anti-mouse CD45 and APC anti-mouse/human CD11b antibodies. CD45^int^/CD11b^high^ labelled cells (corresponding to microglia) were isolated by fluorescence activated cell sorting (FACS) using a SH800Z cell sorter (Sony Biotechnology Inc, Surrey, UK). Sorted microglia were seeded into 24-well plates at a density of 3.0 × 10^4^ cells/well (2.0 × 10^5^ cells/cm^2^) and maintained in fresh DMEM at 37 °C and 5% CO_2_. Experimental procedures involving the handling and use of mice were performed in accordance with institutional animal welfare guidelines and were approved by the state government of Baden-Württemberg, Germany (Regierungspräsidium Tübingen). Mice were housed under a 12 h light/dark cycle and had free access to food and water.

### Fluorescence microscopy

Trypanosome-microglia interaction was initially evaluated microscopically after 2 and 16 h of co-incubation at a 1:30 microglia to parasite ratio. Briefly, after sorting, microglia were allowed to adhere to Millicell® EZ SLIDES (Merck Chemicals GmbH, Darmstadt, Germany) for at least 2 h in DMEM medium without serum. Thereafter, DMEN medium was removed and the TbCMF22-RNAi parasite suspension in HMI-9 medium was added. After different incubation times, medium containing non-adherent cells (trypanosomes and some microglia) was removed, wells were washed twice with PBS. Cells were fixed in 4% paraformaldehyde for 1 h at 4 °C and stained with DAPI for 10 minutes (0.1 mg/ml final concentration). Finally, after removing the grid, slides were covered with Fluoromount G and examined using a Zeiss Z1 AxioObserver inverted fluorescence microscope.

### Flow cytometry

To analyse phagocytosis the standard methodology using flow cytometry was applied^23^. For this purpose, parasites bearing the pT2T7-CMF22RNAi construct were transfected once again at this time with the pCO57 plasmid containing the eGFP gene sequence and the BLE gene that confers resistance to phleomycin under the control of the T7 promotor. Transfection and selection were performed as described before using 2 µg/ml of phleomycin. Fluorescence was controlled microscopically after 72 hours induction with tetracycline. Parasite clones bearing the brightest fluorescent signal were obtained by serial dilution. For the phagocytosis assay microglia were sorted on 24-well plates (30,000 cells per well) and, as described before, allowed to adhere for at least 2 h. Then, pT2T7-CMF22RNAi-GFP parasites were added at ratios 1:5, 1:10, and 1:30 (microglia:parasite). Following different incubation times (from 2 to 20 h) adherent cells, obtained by detaching, and cells found in the supernatant, obtained by centrifugation, were stained with the CD45 and CD11b antibodies and analysed by flow cytometry to quantify triple labelled microglia (CD45^int^/CD11b^high^/GFP^+^).

### Transmission electron microscopy

To analyse the ultrastructure of triple labelled microglia, these cells were sorted on a permeable support PET membrane with 1um size pores (ThinCert™ Cell Culture Inserts, Greiner Bio-One) and prepared for electron microscopy. Fixation was performed in 2.5% (vol/vol) glutaraldehyde (Science Services, Munich, Germany) in 0.2 M sodium cacodylate buffer (Merck, Darmstadt, Germany) at 4 °C overnight. Samples were post-fixed in osmium tetroxide (1%, vol/vol, Serva, Heidelberg, Germany) and stained in 2% uranyl acetate (Science Services, Munich, Germany) in 70% ethanol. After dehydration, membranes were embedded in epoxide resin (Araldite, Serva, Heidelberg, Germany). Finally, blocs were used for ultramicrotomy and sections were stained in 0.4% (vol/vol) lead citrate (Merck, Darmstadt, Germany). Samples were analysed using an EM10 electron microscope (Carl Zeiss, Oberkochen, Germany).

### Nitric oxide determination

Nitric oxide concentrations were determined in the supernatants of axenic cultures (microglia or trypanosomes) and microglia/trypanosome co-cultures, collected during the phagocytosis assay, using the Total Nitric Oxide and Nitrate/Nitrite Assay from R&D Systems by following the manufacture’s recommendations. This commercial kit determines the levels of stable metabolites of NO, nitrite and nitrate, as an indirect measurement of NO. Briefly, after different incubation times (as indicated before), the medium was removed and centrifuged for 10 min at 1200 rpm and 4 °C. Supernatants were immediately frozen and stored at −20 °C until processing. Samples were thawed and deproteinization was performed using 2% zinc sulphate (final concentration). After removal of precipitated proteins by centrifugation (10,000 g, 10 min, at 4 °C), total nitrite was determined indirectly via enzymatic conversion of nitrate to nitrite by nitrate reductase. Culture medium alone was also analysed to subtract the nitrite present, which was not produced by the cells.

### Statistical analysis

Statistical analysis was performed using the GraphPad Software™. Results are shown as mean values ± standard deviations (SD). Statistical significance was calculated by applying Student’s T-test, and indicated as follows: *p < 0.05, **p < 0.01, and ***p < 0.001.
